# Prevalence of Accommodative Insufficiency in Children With Normal Accommodative-Convergence/Accommodation Ratio and Its Association With Refractive Error: A Cross-Sectional Study

**DOI:** 10.7759/cureus.79683

**Published:** 2025-02-26

**Authors:** Shoubhik Chakraborty, Shrutakirty Parida, Soumya k Mohanty, Matuli Das, Shovna Dash

**Affiliations:** 1 Ophthalmology, Kalinga Institute of Medical Sciences, Bhubaneswar, IND

**Keywords:** ac/a ratio, accommodative insufficiency, amplitude of accommodation, prevalence, refractive error

## Abstract

Objective: With the increasing use of digital devices among the pediatric population, asthenopia has become increasingly common. While refractive errors are the most prevalent cause, they can sometimes be linked to undiagnosed or underdiagnosed binocular vision disorders, such as accommodative insufficiency (AI). In many cases, simple refractive correction alone does not alleviate asthenopia. This study aims to determine the prevalence of AI in children with a normal accommodative-convergence/accommodation (AC/A) ratio and to investigate the correlation between accommodative amplitude and refractive errors.

Methods: The cross-sectional study was conducted from April 2024 to December 2024 to assess the prevalence of AI in children aged between six and 18 years with normal AC/A ratios. The study was approved by the Ethics Committee of Kalinga Institute of Medical Sciences, Bhubaneswar, India. The minimum required sample was 384, assuming a 50% prevalence, and was increased to 500 participants to account for a 20% non-response rate. Uncorrected distance visual acuity (UCVA) was assessed using a Snellen chart. Refraction included objective retinoscopy, subjective refraction, and cycloplegic refraction using 1% cyclopentolate. Binocular vision was evaluated using the Hirschberg test, ocular motility assessment, slit-lamp biomicroscopy, intraocular pressure measurement, ophthalmoscopy, and heterophoria testing. The AC/A ratio was calculated following the calculated method. Amplitude of accommodation (AA) was measured using the push-up method with a Royal Air Force (RAF) ruler. Hofstetter’s formula, 15 - (Age/4), was used to determine the minimum expected AA. Participants with AA at least 2.00 D below this threshold were diagnosed with accommodative insufficiency. Participants were categorized into groups of six to 11 years and 12 to 18 years to account for age-related variations in accommodation. Statistical analysis included the chi-square test for categorical comparisons and Spearman's correlation for assessing relationships between AA and refractive error.

Results: Out of the 500 participants, 238 were male and 262 were female, ranging in the age range of six to 18 years, with a mean age of 12.78 ± 3.12 years. It was discovered that 162 individuals (32.4%) lacked adequate accommodations. The mean AA for our study participants was 11.09 ± 2.60 years. The prevalence of AI was 32.4% overall, with 32.6% (95% confidence interval: 27.1-38.4) in females and 32.2% (95% confidence interval: 26.5-38.3) in male patients. We found no discernible relationship between the AA and refractive error in our study.

Conclusion: In this study, with an overall AI prevalence of 32.4%, the ability to maintain comfortable, clear, and effective vision is notably affected by accommodative dysfunction alone, even when AI and convergence insufficiency (CI) coexist. Our investigation found no correlation between refractive error and the AA.

## Introduction

Accommodation is a complex process that enables the eye to shift focus between distant and near objects, ensuring clear vision [1]. This mechanism is regulated by a balance between sympathetic and parasympathetic innervation, allowing precise focus adjustments [2]. Controlled by the preoccipital cortex, accommodation coordinates neural pathways for sharp vision across distances [3]. The dual innervation of the accommodative system enables a faster transition when shifting focus from far to near [4,5]. This adaptive mechanism responds to visual demands through trial and error. Several factors influence accommodation, including object size, target distance, movement, luminance, ocular convergence, refractive errors, and age [6,7].

Accommodation takes longer than convergence due to the latency of the ciliary muscle mechanism [2]. It involves three simultaneous actions, namely, medial rectus contraction, pupil constriction, and ciliary muscle contraction, known as the near accommodative triad [3,8]. Pupillary constriction increases the depth of focus via the parasympathetic nervous system, allowing the lens to steepen for near vision [9]. Vergence aids foveation during focus shifts. The afferent limb detects blurred images, while the efferent limb, including the Edinger-Westphal nucleus and oculomotor neurons, mediates accommodation and convergence [10]. The range of accommodation depends on luminance (cd/m²), as lower levels reduce function due to reliance on cone photoreceptors [11-13].

A study by Abraham et al. reported that myopes had the highest amplitude of accommodation, followed by emmetropes and hyperopes [14]. This principle underlies orthoptic exercises like loose lens rock training, which enhance the amplitude of accommodation (AA) [15,16]. Amplitude of accommodation is crucial for diagnosing accommodative insufficiency (AI), a condition characterized by reduced accommodation ability [17,18]. The relationship between refractive error (D) and AA (D) varies, requiring further research [19].

Our study examines post-cycloplegic refractive error (D) and AA (D) in a pediatric population with a normal accommodative convergence/accommodation (AC/A) ratio (Δ/D). Each participant’s AA (D) is compared with Hofstetter’s minimum amplitude norms (D) to assess deviations [20]. The prevalence of AI in children aged between eight and 16 years is reported as high as 17% [21]. Secondary convergence insufficiency (CI) has been linked to reduced AA (D) [22]. To isolate accommodation effects, our study includes only participants with normal convergence (Δ), ensuring a normal AC/A ratio (Δ/D).

## Materials and methods

Study design

This cross-sectional study was conducted from April 2024 to December 2024 to determine the prevalence of AI in children aged between six and 18 years with a normal AC/A ratio. Participants included individuals presenting with asthenopic symptoms but without an increase in refractive error. The study was designed to assess the association between accommodative amplitude and refractive error while ensuring a controlled evaluation of accommodation function in a pediatric population.

Ethical considerations

The study was approved by the Kalinga Institute of Medical Sciences Ethics Committee, Bhubaneswar, India (approval number: KIIT/KIMS/IEC/1809/2024) and was conducted in accordance with the principles outlined in the Declaration of Helsinki. Informed consent was obtained from the guardians of all participants before enrollment. Participants were assured that their data would remain anonymous and confidential. The study protocol emphasized adherence to ethical standards in participant selection, data handling, and the use of non-invasive optometric assessments.

Inclusion and exclusion criteria

The study included participants aged between six and 18 years who presented with asthenopic symptoms but did not exhibit significant changes in refractive error. Best-corrected visual acuity (BCVA) of 6/6 for distance and N6 for near vision was a mandatory requirement for inclusion. Participants were required to have a normal AC/A ratio as determined by standard clinical procedures.

Exclusion criteria included participants with BCVA <6/6 in either eye, the presence of amblyopia, strabismus, or any history of ocular/head trauma, or previous ocular surgeries. Individuals who were using systemic or ocular medications known to affect accommodation were also excluded. Participants with subjective refraction differing by more than ±0.50 D from their habitual correction were excluded from the study to ensure that accommodative function was assessed independently of significant refractive changes [6].

Study procedure

Each participant underwent a comprehensive optometric examination, including the assessment of visual acuity, refractive error, and binocular vision function. Uncorrected distance visual acuity (UCVA) was measured using a Snellen chart at six meters. Objective refraction was performed using a static retinoscope, followed by subjective refraction to refine the prescription. Cycloplegic refraction was performed using 1% cyclopentolate ophthalmic solution, followed by wet retinoscopy to ensure accurate refractive measurements without accommodative interference. A complete ocular examination was conducted, including the Hirschberg test to evaluate ocular alignment, ocular motility assessment, slit lamp biomicroscopy for anterior segment evaluation, intraocular pressure measurement using a non-contact tonometer, and ophthalmoscopy to rule out any posterior segment pathology. Heterophoria magnitudes were assessed using the prism bar cover test and Maddox rod at 40 cm (N8 target) for near and 6 m (6/9 target) for distance. The AC/A ratio was calculated following Scheiman’s recommendations [17].

Tools and assessments

Amplitude of accommodation was measured using the push-up method with a Royal Air Force (RAF) ruler. A small N8 target was moved closer to the participant’s eye until a sustained blur occurred. The AA value in diopters (D) was calculated using the formula AA (D) = 100/NPA (cm), where NPA represents the near point of accommodation. The NPA was measured three times for each eye individually and binocularly, with two-minute intervals between each measurement to account for any variability in accommodative response.

The right eye's AA values were used for analysis, as monocular measurements provide a more accurate estimate of accommodative function without the influence of binocular cues [21]. The AC/A ratio was determined using the calculated method, comparing phoria measurements at a distance (6 m) and near (40 cm). The equation applied was AC/A Ratio = (Stimulus Accommodation (D) × (Near Phoria - Distance Phoria)) + (Near Fixation Distance in meters × Interpupillary Distance in cm). This method accounts for the relationship between vergence movements and accommodation, considering interpupillary distance (IPD) and fixation distance. A normal AC/A ratio is typically between 3:1 and 5:1 [6].

Testing conditions were standardized, with all assessments performed under controlled lighting of 650 cd/m². All examinations were conducted by a trained pediatric optometrist to ensure consistency and accuracy in data collection. Participants were excluded from further analysis if their BCVA was <6/6, they had amblyopia or strabismus, or if they had systemic or ocular conditions that could influence accommodation [6].

Sample size calculation

The minimum sample size required for this study was determined based on an estimated AI prevalence of 50%. Using this assumption, a minimum of 384 participants was needed to achieve sufficient statistical power. To account for a 20% non-response rate, the adjusted sample size was calculated using the formula: Adjusted Sample Size = 384 / 0.80 = 480. To further enhance the reliability and generalizability of the study, the sample size was rounded up to 500 participants. A larger sample improves precision in estimating prevalence rates, allows for better subgroup analysis, and increases statistical power to detect potential associations between accommodative function and refractive error.

Statistical analysis

Descriptive statistics were used to calculate the prevalence of AI among the study population. The chi-square test was employed to analyze associations between categorical variables, such as prevalence rates across different age groups and gender. This test is particularly useful for comparing the frequency distributions of AI among subgroups within the population. The Spearman's correlation test was used to assess the linear relationship between refractive error and AA. Spearman's correlation is suitable for evaluating numerical trends and determining the degree of association between two continuous variables. A p-value of <0.05 was considered statistically significant, ensuring that any observed relationships between refractive error and accommodative function were unlikely to be due to chance.

## Results

The study examined the demographic distribution of participants with AI and those with normal accommodation, based on the difference between minimum (Min AA) and present AA (Present AA). The mean age of the total sample was 12.78 ± 3.12 years, with the normal accommodation group having a mean age of 12.61 ± 3.23 years and the AI group having a higher mean age of 13.43 ± 2.78 years. A chi-square test indicated a statistically significant difference in age distribution between the normal accommodation and AI groups (χ² = 2.771, p = 0.006), suggesting that older individuals were more likely to have AI.

The prevalence of AI was 32.4% overall. Age group analysis showed that among participants aged between six and 11 years (n = 229), 72.1% (n = 165) had normal accommodation, while 27.9% (n = 64) had AI. In the 12-18 years group (n = 271), the prevalence of AI increased to 36.2% (n = 98), with 63.8% (n = 173) having normal accommodation. Although the chi-square test for age groups approached significance (χ² = 3.824, p = 0.051), it did not reach the conventional threshold, indicating a potential trend of increasing AI prevalence with age.

Regarding gender distribution, there were 261 female participants, of whom 67.4% (n = 176) had normal accommodation and 32.6% (n = 85) had AI. Among 239 males, 67.8% (n = 162) had normal accommodation, while 32.2% (n = 77) had AI. The chi-square test showed no statistically significant difference in AI prevalence between genders (χ² = 0.007, p = 0.934). Regarding gender, among 261 female participants, 176 (67.4%) had normal accommodation, while 85 (32.6%) had AI. Among 239 male participants, 162 (67.8%) were in the normal group, and 77 (32.2%) had AI. The chi-square value for gender was 0.007 (p = 0.934), showing no significant difference between male and female participants. All the above findings are presented and summarized in Table 1. 

**Table 1:** The prevalence of accommodative insufficiency (AI) by age and gender Min AA: minimum amplitude of accommodation; Present AA: present amplitude of accommodation

Demographic parameters		Total	Normal accommodation group		Accommodative insufficiency group		Chi-square	p-value
			(Min AA-Present AA) <2.00		(Min AA-Present AA)>=2.00			
			Count	Percentage	Count	Percentage		
Age (years)		12.78±3.12	12.61±3.23		13.43±2.78		2.771	0.006
Age group (years)	6-11	229	165	72.1%	64	27.9%	3.824	0.051
	12-18	271	173	63.8%	98	36.2%		
Gender	Female	261	176	67.4%	85	32.6%	0.007	0.934
	Male	239	162	67.8%	77	32.2%		

Table 2 presents the 95% CIs for gender and refractive error, offering a clearer estimate of the potential variation in AI prevalence within these groups. Table 2 provides additional insights by demonstrating that refractive error has no significant impact on AI prevalence, as evidenced by the overlapping CIs. These findings reinforce the notion that age may be a contributing factor, while gender and refractive error do not appear to play a significant role in AI occurrence.

**Table 2:** The prevalence of accommodative insufficiency (AI) by age, gender, and refractive errors CI: confidence interval; 95% CI was calculated using a binomial distribution

Category		Accommodative insufficiency group % (95% CI)
Gender	Male	32.6% (27.1 - 38.4)
	Female	32.2% (26.5 - 38.3)
Age group (years)	6-11	27.9% (22.4 - 34.0)
	12-18	36.2% (30.6 - 42.0)
Refractive error	Emmetropia	32.8% (27.7 - 38.2)
	Myopia	31.8% (25.6 - 38.5)

**Figure 1:**
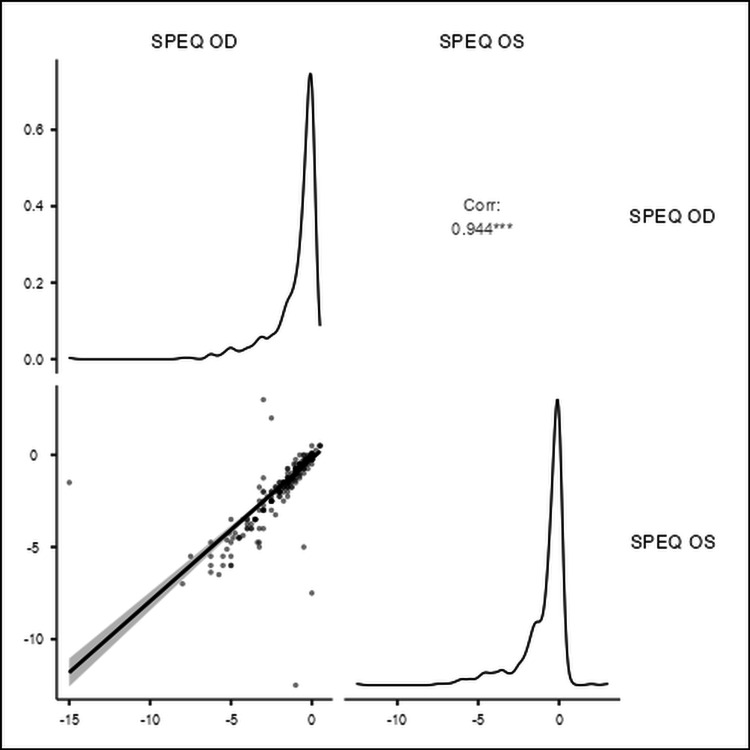
The correlation (Corr) between spherical equivalent of both eyes SPEQ OD: spherical equivalent of the right eye; SPEQ OS: spherical equivalent of the left eye

Figure 1 presents Spearman's correlation test that showed a strong positive correlation (r = 0.944) between the spherical equivalent values of the right and left eyes.

**Figure 2:**
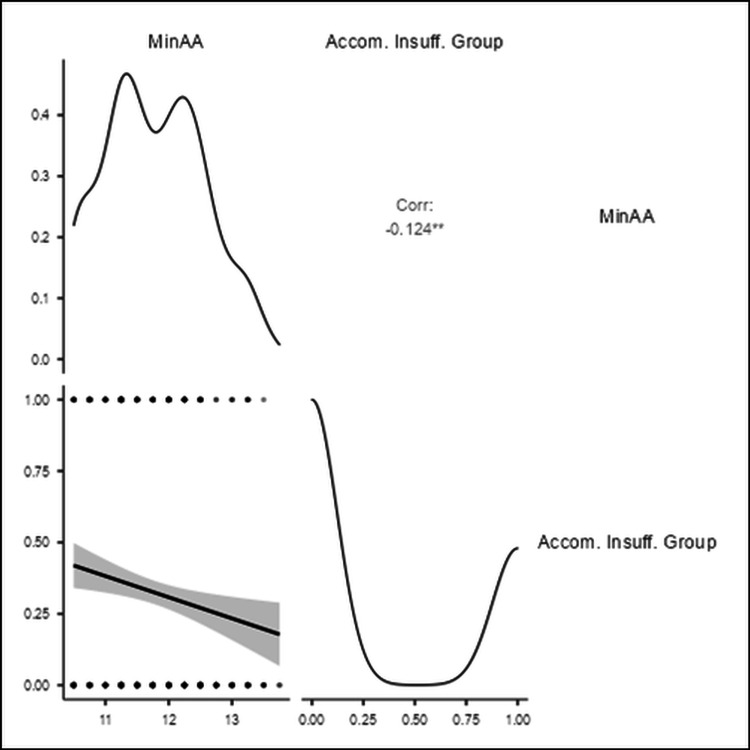
The correlation (Corr) between minimum amplitude of accommodation (Min AA) and accommodative insufficiency (Accom. Insuff.) group

However, the Min AA demonstrated a statistically significant negative correlation (r = -0.124) with the AI group shown in Figure 2.

No significant correlation was found between the current AA and refractive error.

## Discussion

Binocular vision disorders affect approximately 30% to 34% of the population [23, 24]. The increasing prevalence of convergence and accommodation disorders has been largely attributed to excessive use of digital devices, particularly for near tasks. In this study, we examined the prevalence of AI and its relationship with refractive error and demographic parameters in an Eastern Indian population aged between six and 18 years. Previous studies have reported AI prevalence rates ranging from 1% to 61.7%, with a median value of 31.35%, which closely aligns with our study’s prevalence of 32.4%.

Children with binocular vision disorders often experience emotional and social challenges and may sometimes be misdiagnosed with learning disabilities, such as dyslexia [25]. The prevalence of AI is expected to increase further following the COVID-19 pandemic due to prolonged screen time across all age groups [23]. Given the rising incidence of CI, our study is particularly relevant in the modern digital era.

Among the three diagnostic methods for evaluating the AA which are the push-up method, pull-away method, and minus lens blur method, the push-up method remains the most widely used in both adults and children [26]. In this study, we analyzed AI prevalence in relation to age, gender, and refractive error, but none of these factors showed a statistically significant correlation. Our findings align with those of Lynn et al., who reported no significant effect of gender on AI prevalence [27]. Similarly, a population-based cross-sectional study by Hashemi et al. in the Iranian population found no statistically significant association between AI prevalence and refractive error [28].

In contrast, a study by Abdul-Kabir et al. on junior high school students reported an AI prevalence of 39%, a rate comparable to our findings [29]. The primary diagnostic criterion for AI across studies is a measured AA at least two diopters below Hofstetter’s minimal age-expected amplitude formula [30]. Convergence insufficiency is characterized by a low accommodative convergence to AC/A ratio, with increased near exophoria relative to distance phoria. To rule out convergence-associated accommodative insufficiency (CAAI), we included only participants with a normal AC/A ratio in our study.

Convergence-associated accommodative insufficiency results in difficulty maintaining focus while converging on near objects, leading to blurred vision, eye strain, and reading difficulties. It can be influenced by prolonged near work, uncorrected refractive errors, neurological conditions, medications, and systemic diseases. A normal AC/A ratio typically ranges from 3:1 to 5:1, meaning the eyes converge 3 to 5 prism diopters per 1 diopter (D) of accommodation [31]. A high AC/A ratio (>5:1) is associated with convergence excess (CE), while a low AC/A ratio (<3:1) is linked to CI. This was assessed in our study using the calculated method. Maintaining a normal AC/A ratio is essential for comfortable binocular vision and efficient focusing [31].

Pseudo-accommodative insufficiency (PAI) is also associated with a high AC/A ratio [32]. To minimize the influence of vergence-related disorders on our findings, we excluded participants with conditions that could affect binocular vision stability. Vergence-related disorders that influence accommodation include CI and CE, which primarily affect near vision and divergence insufficiency (DI), and divergence excess (DE), which affect distance vision. Both DE and CE are usually associated with a high AC/A ratio, while CI and DI are linked to a low AC/A ratio. These conditions can be influenced by uncorrected refractive errors, prolonged near work, neurological conditions, aging, medications, and poor binocular vision development.

Fusional vergence dysfunction (FVD) cannot be entirely ruled out, as it presents with a normal AC/A ratio. FVD is a binocular vision disorder in which the eyes struggle to maintain alignment due to impaired fusional vergence, leading to symptoms such as eye strain, headaches, and difficulty focusing, particularly during prolonged near work. Unlike AI, FVD does not affect accommodation but instead results in poor convergence and divergence control.

A key limitation of our study was the lack of accommodative facility testing, which assesses how efficiently the eyes shift focus between distances. Accommodative facility testing involves the accommodative flipper test (±2.00 D) and Near Rock Card, which measures response speed in shifting focus [33]. This test is useful for diagnosing AI but was excluded due to its complexity for younger children. Our study’s mean AA of 11.09 (±2.60) D aligns with findings from Hussaindeen et al. [34]. A key limitation is its single-center design, limiting generalizability to the broader Eastern Indian population. Relying solely on the AA may not capture all accommodative dysfunctions. Future research should include multi-center studies and additional diagnostic tests for a more comprehensive evaluation of AI.

## Conclusions

Our study found a 32.4% prevalence of AI among 500 participants aged between six and 18 years, with similar rates in male and female participants. The mean AA was 11.09 ± 2.60, with no significant correlation to refractive error. Accommodative dysfunction alone can impair visual comfort and efficiency, particularly as digital device use increases, placing greater demand on near vision tasks. Early detection and timely intervention are crucial to preventing long-term vision-related challenges, enhancing academic performance, and improving overall quality of life. Regular vision screenings and appropriate management strategies are essential for maintaining optimal eye health in children and adolescents.
